# Peer assisted learning among Sri Lankan medical undergraduates: a cross sectional study

**DOI:** 10.1186/s13104-017-2920-2

**Published:** 2017-11-14

**Authors:** Nipun Lakshitha de Silva, Balasundaram Parththipan, Chaturaka Rodrigo, Godwin Constantine, Sumadhya Deepika Fernando, Senaka Rajapakse

**Affiliations:** 1 Department of Clinical Medicine, Faculty of Medicine, University of Colombo, 25 Kynsey Road, Colombo 08, Sri Lanka; 2 School of Medical Sciences, Faculty of Medicine, University of New South Wales, Sydney, NSW 2052 Australia; 3 Department of Parasitology, Faculty of Medicine, University of Colombo, 25 Kynsey Road, Colombo 08, Sri Lanka

**Keywords:** Peer-assisted learning, Undergraduate teaching, Medical students, Sri Lanka

## Abstract

**Objective:**

The objectives of this study were to; (a) evaluate the current practices of peer assisted learning among second year and final year medical students of Faculty of Medicine, University of Colombo, Sri Lanka; (b) identify reasons for engaging in peer assisted learning; (c) identify perceived weaknesses in current learning activities; and (d) determine student characteristics associated with engaging in peer assisted learning.

**Results:**

This cross sectional study interviewed two hundred and eighty-four eligible students. Average number of hours spent on peer assisted learning during a week was significantly greater among second year students compared to final year students (15.1 vs. 7.1 h, p < 0.05). Overall, female students were more likely to engage in peer assisted learning than male students. In second year, most common method of peer assisted learning was mass lectures offered by batch mates or seniors, while in final year it was group discussions. This reflected a transition to more focused, interactive, active learning among senior students.

## Introduction

Peer assisted learning (PAL) refers to an act or process of gaining knowledge or skills from other students who are at either different or equivalent academic or experiential levels, through instruction or experience [1]. The term ‘peer’ can be further specified as co-peers, who are at the same academic or experiential level, and near-peers, who have already surpassed the level taught [2]. PAL is a widely recognized method of learning used by medical undergraduates in both clinical and class room settings [3].

The General Medical Council, UK, recommends that all medical students should be trained as teachers [4], since one of the responsibilities of medical professionals is teaching and training. In Sri Lanka however, opportunities for medical students to become formal peer teachers are limited and at times, discouraged. Nonetheless, PAL exists informally among medical students.

It has been suggested that PAL can potentially achieve equivalent learning outcomes compared to conventional teaching [5]. There is also evidence to show that participating student teachers benefit academically and professionally [5]. Both teaching and preparing to teach has a positive impact on the learning processes of peer teachers [6]. Further, there is evidence to suggest that medical students are capable of organizing quality PAL programmes, and that feedback from peer students can be used to further improve these programmes [7]. The enthusiasm and interest of both the peer teacher and the student, makes knowledge transfer via PAL more effective [8]. There are frameworks designed to help planning and implementing medical undergraduate PAL programmes. These help to formalize it as a mode of teaching in medical curricula [9].

Published evidence on PAL of medical undergraduates in Sri Lanka is limited. There is a need to understand the utilization and acceptance of PAL in the Sri Lankan context, with the goal of implementing a formal teaching structure for PAL within medical school curricula. This would benefit students, while minimizing the limitations of peer teaching such as dissemination of outdated or incorrect information. Identifying reasons that motivate medical students to seek PAL would also help to identify deficiencies in the formal curriculum, which could be corrected by reflective practice.

The objectives of this study were to (a) evaluate the current practices of PAL among second year and final year medical students; (b) identify the reasons to engage in PAL; (c) identify perceived weaknesses in current PAL activities; and (d) determine characteristics of students engaging in PAL.

## Main text

### Methods

A cross-sectional descriptive study was conducted in the Faculty of Medicine, University of Colombo in September 2013, with the participation of second and final year medical students. Second year students were selected as they had just completed the Introductory Basic Sciences Stream (IBSS) examination which is the first barrier examination in the faculty. A hype of PAL occurs during the preparatory phase for this examination, and we hypothesized that those who had recently engaged in PAL would recall their experience better. Final year students have completed most of the undergraduate medical curriculum and are therefore in a position to give an overall feedback on PAL activities during their undergraduate career.

Data was collected using a self-administered questionnaire designed by the investigators following an extensive literature search. This was pre-tested on ten third year students. The questionnaire covered socio-demographic details, information on current practices of PAL, reasons for seeking PAL, and perceived strengths and weaknesses of PAL. To ensure consistency in answers, most questions were kept as multiple choice questions with one answer to select from. However, there was an option for students to write the answer if the most suitable answer did not appear in the list provided. Certain questionnaire items such as usefulness of PAL was scored with a Likert scale from 1 to 5 with one being “least useful” and 5 being “most useful”. All students in the selected batches were invited to participate and the volunteers were enrolled into the study after informed consent.

Data was entered and analyzed using Statistical Package for Social Sciences (SPSS) version 19^®^ [IBM corporation, New York, USA]. Descriptive statistics are given as frequencies, proportions, percentages and summarized with means plus standard deviation. Statistical significance of association between dichotomous variables was analyzed using Chi square test and that of continuous variables with independent sample t test.

### Results

#### Socio-demographic data

Out of 352 invited, 284 students (80.7%) participated. This included 162 second year students (females; 56.8%) and 122 final year students (females; 43.4%). The language of instruction of the undergraduate medical curriculum is English only. However, the native languages of Sri Lanka are Sinhalese and Tamil. Of the second year students, 76.9% spoke Sinhala as their first language and 12.6% were conversant in Tamil. Of the final year group, 82.5% spoke Sinhalese and 10.7% spoke Tamil. All students had undergone an intensive training programme in English prior to the commencement of the undergraduate course.

#### Patterns of utilization of PAL

The overall frequency of utilization of PAL when scored in a Likert Scale from 0 to 5 did not vary significantly between second year (3.24) and final year (3.07) students. The same was true of the frequency of using PAL during term or just before examinations. However, during term, second year students spent approximately double the number of hours on PAL compared to final year students (15.07 vs. 7.12 h per week, p = 0.002). During last two weeks prior to an exam average time spent for PAL was 11.98 h among second years and 7.84 h among final year students (p < 0.05). Only four (2.5%) second year students and seven (5.7%) final year students reported to have never participated in PAL.

Majority of second year students (101/126, 80.16%) participated in PAL activities conducted by co-peers (i.e., batch-mates) rather than by near-peers (senior students). In contrast, only 43% (29/68) of final year students selected a batch-mate as the first choice for a peer teacher (p < 0.001). For second year students, the preferred method of PAL was mass lectures (57/114, 50%), while the final year students preferred group discussions (20/66, p < 0.001).

The medical curriculum in Faculty of Medicine, Colombo has 14 subjects (Anatomy, Biochemistry, Physiology, Pathology, Microbiology, Parasitology, Pharmacology, Community Medicine, Forensic Medicine, Clinical Medicine, Surgery, Paediatrics, Gynaecology and Obstetrics, Psychiatry) taught in activities compacted into five streams (Introductory Basic Sciences Stream, Applied Sciences Stream, Community (Public Health) Stream, Behavioural Sciences Stream and Clinical Sciences Stream). Participants were asked to indicate the streams and subjects that they prefer to use PAL to supplement the formal teaching. For final year students chose Behavioural Sciences Stream (40/122, 32.8%) and Clinical Sciences Stream (32/122, 26.2%). The second year students who had only been taught within the Introductory Basic Sciences Stream, selected Anatomy (29/54, 53.7%), followed by Physiology (20/54, 37%) and Biochemistry (3/54, 5.6%) as the subjects needing supplementary teaching via PAL. Other aspects of PAL as reported by the participants are summarized in Table 1.

**Table 1:** Comparison of prior preparation and after studying in relation to PAL among second year (n-162) and final year (n-122) students

	Second year (%)	Final year (%)	Chi square value
Preparation			
Any kind of preparation	123 (82.6%)	69 (59%)	22.541*
Read relevant lecture notes	114 (75.5%)	50 (42.7%)	32.915*
Read relevant sections in text books	86 (57.3%)	52 (44.4%)	8.180
Refer to internet	54 (37.7%)	27 (23.1%)	9.625*
Make own notes	44 (29.1%)	24 (20.5%)	3.549
After studies			
Any kind of after studies	146 (96.1%)	105 (89.7%)	4.826
Read relevant lecture notes	115 (75.7%)	75 (64.1%)	4.867
Read relevant sections in text books	117 (77%)	82 (71%)	2.233
Refer to internet	62 (40.8%)	45 (38.5%)	0.749
Make own notes	75 (45.3%)	55 (47%)	0.743
Refer to notes made during the PAL	74 (48.7%)	55 (47%)	0.249

Different methods of preparatory work by students prior to PAL activities and after studies following PAL activities are indicated with percentage. Since response rate for each question is less than 100% percentage calculated based on the number of responders as the denominator

* p < 0.05

For many second year students (132/154, 85.7%), the primary reason to engage in PAL was exam preparation. Interestingly, final year students mentioned that gaining in-depth knowledge of the subject area (102/116, 87.9%) as well as exam preparation (101/116, 87.1%) as important reasons for PAL (Table 2). A significant proportion of students (121/267, 45.3%) mentioned that exam oriented nature of PAL activities was in fact a weakness (Table 2).

**Table 2:** Self-reported strengths and weaknesses of PAL: a comparison between first year (n-162) and final year (n-122) students

	Second year (%)	Final year (%)	Chi square value
Strengths			
Preference for small group learning	116 (75.3%)	72 (62.1%)	5.498
Room to clarify doubts	118 (76.6%)	74 (63.8%)	5.302
Convenient, flexible schedule	59 (38.3%)	48 (41.4%)	0.260
Easier to concentrate	84 (54.5%)	67 (57.8%)	1.701
Use of native language	95 (61.7%)	65 (56%)	0.876
Help with exam preparation	132 (85.7%)	101 (87.1%)	0.103
Easy to understand peer teaching	99 (64.3%)	60 (51.7%)	4.313
Supplements formal teaching	114 (74%)	79 (68.1%)	1.139
Non-threatening environment	118 (76.6%)	89 (76.6%)	0.000
Summarizes important points	119 (77.3%)	94 (81%)	0.562
Providing in-depth knowledge of the subject area	116 (75.3%)	102 (87.9%)	6.762*
Weaknesses			
Reliability of content	62 (40.8%)	39 (33.6%)	1.648
Facts being unaccepted in examinations	63 (41.7%)	43 (37.1%)	1.028
Promotion of exam oriented learning	70 (46.4%)	51 (44%)	0.585
Irrelevant content	51 (33.6%)	15 (13%)	14.831*

Strengths and weaknesses of PAL activities as indicated by the students with percentages presented in table

* p < 0.05

Further analysis was performed by combining both student groups to identify characteristics associated with frequent utilization of PAL. Male students, those doubted the authenticity of information taught by peers and those who disliked strategic learning approach of PAL were significantly less likely to engage in PAL activities (Table 3). Interestingly, the medium of instruction during school education (either Sinhalese or Tamil) did not influence the frequency of utilization of PAL (p = 0.766).

**Table 3:** Association of student characteristics with mean self perceived frequency of participation in PAL (Likert scale 0–5)

Characteristic	Categories	Mean frequency	t test
Gender	Male	2.892	− 2.964*
	Female	3.422	
Medium of school education	English	3.231	0.183
	Native	3.159	
Attempt at which entered medical school	First	3.050	− 1.393
	Second or third	3.316	
Student having doubt about veracity of content	Yes	2.870	− 2.518*
	No	3.346	
Student believing that PAL promotes exam oriented learning	Yes	2.905	− 2.716*
	No	3.401	
Performing preparatory work prior to attending to PAL	Yes	3.099	− 1.507
	No	3.393	
Performing after studies following PAL	Yes	3.312	3.917*
	No	2.059	

Some likely factors to associate with frequency of utilization of PAL are given in the table. Students’ perceived weaknesses also negatively affect the participation in PAL

* p < 0.05

### Discussion

The Faculty of Medicine, University of Colombo is the oldest and largest medical school of the country. This institution incorporated large scale reforms into the curriculum in 1994, changing the subject based curriculum to an integrated curriculum of five streams with more student centered learning activities. Despite the embracing of student centered learning and evidence for its usefulness, PAL has not been recognized as a formal method of learning by the faculty [8, 10, 11]. There are many concerns regarding PAL among staff teachers as it can be a source of misinformation. It also may encourage strategic learning as opposed to deep learning. Nevertheless, in our experience as past students and current teachers of the same faculty, PAL is frequently used by students to supplement formal teaching. Characterizing the nature, quality and reasons for which students pursue PAL, would help to negotiate its inclusion into formal teaching to benefit all parties concerned.

According to the observations of this study, PAL was more popular among second year students than final year students. Nevertheless, in both groups, a majority were involved in PAL activities. Some reasons for this discrepancy are obvious. Final year students have a busy clinical schedule and there is less time to engage in PAL in addition to formal teaching. Also, if engaging in PAL, they have to do with peers of the same batch instead of more experienced seniors. When progressing to the final year, medical students tend to seek more group based PAL activities, which could be considered a positive trend. Formal group teaching activities such as small group activities and problem-based-learning sessions cannot be held for every core topic due to limited resources and this gap may be partially filled by PAL.

The second year students took their PAL activities more seriously and engaged in pre-preparation more than the final year students. It was also noted that the internet was inadequately utilized for preparatory work or after-studies in PAL. A previous study in the same institution has shown that students have adequate access to internet, thus this is unlikely to be due to lack of internet facilities [12]. It is interesting to explore whether the content taught by peer teachers is limited to the knowledge accrued from standard textbooks.

A study conducted in another medical faculty in Sri Lanka has looked at the usefulness of PAL from a qualitative approach [13]. In this study, the most cited importance of PAL was to fill gaps in understanding of formal teaching. This matches with the findings of this study, and the quantitative methodology used in our study has made it possible to look at a larger sample size. Since most of the areas addressed in our study have not been studied in recent literature, these findings can provide a background for further studies as well as serve as evidence for the case of integrating PAL activities into formal teaching.

Finally to summarize the conclusions and recommendations from this study;

- Students spend a substantial amount of time per week on PAL activities despite it not being on the standard curriculum.
- PAL was an important source of knowledge acquisition within the sample studied.
- Curriculum developers should pay attention to integrate PAL into the medical curriculum.
- Academic staff should be advised to incorporate PAL activities into their standard teaching schedule to enhance student learning.
- PAL should be seen as an opportunity that can be utilized to enhance active learning by students.

## Limitations

This study was of a cross sectional design. If the same cohort of students were followed up during their stay in medical school, the change in PAL activities could have been appreciated with better validity. The response rate was poor in the final year group due to their busy schedule. For different questions of the study tool response rates were different. When the data collection tool is lengthy and complex responders might tend not to respond all questions particularly if it takes time to respond. A qualitative focus group discussion based study can address this shortcoming.

## Abbreviations

PAL: peer assisted learning
SPSS: Statistical Package for Social Sciences
